# *Salvia officinalis L.* induces alveolar bud growing in adult female rat mammary glands 

**Published:** 2015

**Authors:** Malihezaman Monsefi, Mehrnaz Abedian, Zahra Azarbahram, Mohammad Javad Ashraf

**Affiliations:** 1 *Biology Department, College of Sciences, Shiraz University, Shiraz, Iran*; 2 *School of Medicine, Shiraz University of Medical Sciences, Shiraz, Iran*

**Keywords:** *Alveolar buds*, *Salvia officinalis*, *Lobules*, *Whole mount mammary gland*

## Abstract

**Objectives:**

In traditional medicine *Salvia officinalis *(sage) has been used as menstrual cycle regulator. In the present study the effects of sage extract on breast tissue were examined.

**Materials and Methods:**

Fourteen female rats were divided into two groups: 1) Distilled water-treated rats (Con) that were gavaged with 1ml distilled water and 2) *Saliva officinalis *hydroalcoholic extract (SHE)-treated rats that were gavaged with 30mg/kg/body weight of sage extract for 30 days. The estrus cycle changes were monitored by daily examination of vaginal smear. Whole mounts of right pelvic breast were spread on the slide and stained by carmine. The number of alveolar buds (ABs) type 1 and 2 and lobules of mammary gland were scored. Tissue sections of left pelvic mammary gland were prepared and its histomorphometrical changes were measured. Blood samples were taken from dorsal aorta and estradiol and progesterone concentrations were measured using radioimmunoassay.

**Results:**

Estrous cycles decreased significantly in SHE-treated animals. The number of alveolar buds and lobules in mammary gland whole mount of SHE-treated group were higher than the Con group. The number and diameter of ducts in histological section of mammary gland in SHE-treated group increased as compared to the Con group.

**Conclusion:**

Sage promotes alveologenesis of mammary glands and it can be used as a lactiferous herb.

## Introduction

A mammary gland is an apocrine gland that consists of alveoli lined with simple cuboidal epithelium surrounded by myoepithelial cells. These alveoli join to form groups known as lobules. Each lobule has a lactiferous duct that drains into openings in the nipple. Maintenance of the morphology of the lactiferous duct tree requires extracellular matrix (ECM) such as adipocytes, fibroblast, inflammatory cells, etc. that constitute mammary stroma (Watson and Khaled, 2008 ▶).  Lactiferous duct development occurs in females in response to circulating hormones. Estrogen promotes ductal branching differentiation (Sternlicht, 2006 ▶) by stimulation of estrogen receptor alpha (ER-α) and progesterone receptor (PR) in ductal epithelium. Once ER is activated by estrogen, it is able to translocate into the nucleus and bind to DNA to regulate the activity of different genes and induce biological effects.

In rats, the nipples and mammary glands develop along the two milky lines, two nearly parallel lines along the ventral side of the body. Mammary glands are in pairs along these lines. There are six pairs of nipples including three pairs in anterior (thoracic) region, two pairs in intermediate (abdominal) region and one pair in posterior (inguinal) region of the body. Male rats do not have nipples.

*Salvia officinalis* (sage), a plant belonging to the Lamiaceae family, chiefly contains monoterpenes such as α and β-thujone, α and β-pinene and camphor. In addition, *Salvia officinalis ) S. officinalis(* leaves contain cineol, rosemaric acid, tannic acid, bitter substances like cornsole and cornsolic acid, fumaric, chlorogenic, caffeic and nicotinic acids, nicotinamide and flavones. Sage has a long history of medicinal and culinary use (Hamidpour et al., 2014 ▶). Anti-inflammatory effects of ursolic acid of sage were reported (Baricevic, 2001 ▶). Sage tea has been traditionally used in the treatment of bronchitis, cough, asthma, angina, inflammations of mouth and throat, depression, excessive sweating, and skin diseases (Walch et al., 2011 ▶; Khan et al., 2011 ▶). Sage essential oil has been reported to have carminative, antiseptic, antispasmodic properties. It is effective in the treatment of diseases of nervous system, circulatory system, and respiratory system (Loizzo et al., 2007 ▶). Sage tea increased anti-oxidant potential of hepatocytes by increasing glutation-s transferase and glutation reductase activity (Lima, 2005 ▶). Anti-microbial activity of essential oil of *S. officinalis* against *Staphilicocos aureous* and *Candida albicans *were explained (Alizade and Shaabani, 2012 ▶). Considering terpenoid and flavonoid content of sage (known as phytoestrogens), we hypothesized that this herb could increase mammary gland alveologenesis therefore, sage potential of alveolar bud growing was studied.

## Materials and Methods

**Preparation of extracts**

*S. officinalis *(sage) leaves were obtained from Zardband Pharmaceuticals Company, Tehran, Iran. Sage leaves were powdered, and 100 g of powder was percolated with 800 ml of 70% ethanol for three days to obtain hydroalcoholic extracts. Subsequently, the mixtures were filtered and concentrated under reduced pressure using a rotary evaporator and vacuumed desiccator. The yield (w/w) of extraction method was 17% (g/g). 

**Animal grouping**

Female Wistar rats weighing between 150 to 200 g were obtained from Animal House of Razi Institute, Shiraz, Iran. The animals were adapted to the laboratory conditions for two weeks prior to the experiments. The rats were kept at a controlled temperature (22-24°C) and a 12:12 h light-dark cycle (lights on 6:00; lights off 18:00) with free access to food and tap water. Animal experiments were approved by the Institutional Animal Ethics and Health Committee of the Biology Department of Shiraz University, and were performed according to the principles of the care and use of laboratory animals established by the National Institute of Health. Vaginal smears were examined daily and normal estrous cycle female rats were selected. Estrous cycle lasted 4-5 days and was divided into three

phases of proestrus (9-16 h), estrus (9-16 h) and diestrus (60-70 h) (Montes and Luque, 1998 ▶). Female rats with regular estrous cycles were randomly divided into two groups (n=7) of control (Con) and sage hydroalcoholic extract (SHE)-treated rats that were gavaged with 1 ml distilled water and 30mg/kg/body weight of sage extract for 30 days, respectively. 

**Hormonal assay**

At the end of the experiment, day 30, animals that were in estrus phase of estrous cycle were dissected under deep anesthesia and blood samples were taken from dorsal aorta and centrifuged at 2000 rpm for 20 min. Estradiol and progesterone concentrations were measured using radioimmunoassay (RIA). For this purpose, serum samples were transferred to Department of Hormonal assay, Research Center of Namazi Hospital, Shiraz, Iran.

**Mammary gland whole mount preparation**

**Figure1.**
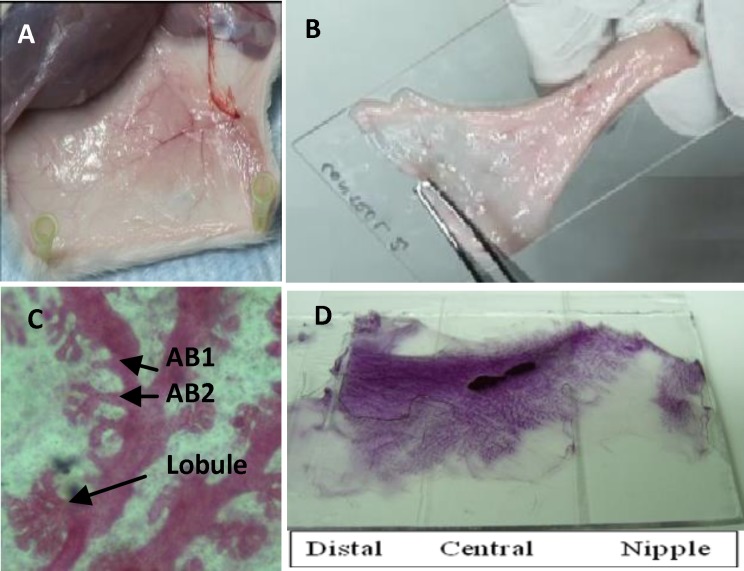
Mammary gland whole mount preparation. Rats were dissected and mammary glands were exposed (A). Pelvic mammary gland was separated and spread on glass slide (B). (D) Mammary gland whole mount slide was divided to three parts of distal (fat pad), central (around lymph node or dark mass) and nipple (tip of breast). Alveolar buds (AB1 and AB2) and lobules of whole mount were demonstrated after carmine staining (C).

At the end of 30-day experiments, rats from each group were dissected and the right mammary gland of pelvic region, free from skin, were removed from area close to the nipple towards the distal end of the gland. It was spread immediately onto a labeled glass slide, fixed with Carnoy's fixative (75% glacial acetic acid and 25% absolute ethanol) at room temperature (RT) for two days. Slides were washed with 70% ethanol for 1 h and distilled water for 30 min. Then, slides were stained with carmine alum and left for two days. Excess dye was washed using increasing concentrations of ethanol (70, 95, 100%) (slides were left in each concentration of alcohol for 1 h) and cleared with xylene and left at RT at least for two days. Mammary gland whole mounts were examined under light microscope (Figure 1) and the number of alveolar bud type 1 (AB1), alveolar bud type 2 (AB2) and lobules (L) were recorded and scored from 0 to 5 in 15 microscopic fields for each animal. AB1 AB2 and lobule consisted of one or two small buds, more than two buds and a cluster of buds on a duct respectively (Figure 1-C). Whole mounts were divided into three parts of nipple area (tip of breast), central zone (around the lymph node) and distal area or near to the fat pad (Figure 1-D). Five microscopic fields were examined in each part. Mammary gland differentiations were assessed in two ways: First, the score values of AB1+AB2+L (Dif1) and second, the ratio between L and AB1+AB2 scores (Dif2). ABs differentiated to lobules, therefore, the higher ratio of lobules/ABs (Dif2) represented more differentiated mammary glands.

**Histomorphometrical studies**

Left pelvic mammary glands of rats were removed and fixed in 10% buffered formalin solution for one week. Then, paraffin blocks were prepared (**Bancroft and Steven, 1991**) from which 6 µm-thick sections were stained with haematoxylin-eosin. Histomorphometric studies examined 5 microscopic fields for each section (3 microscopic slides for each animal). The number of ducts, lobules, branch of ducts and duct with deposits were recorded. Then, ductal and its epithelial and stromal diameters were measured using an ocular micrometer (Zeiss, Germany). 

**Statistical analysis**

Statistical analyses were done using SPSS version 17.5 software. Data were analyzed using one-way ANOVA, followed by Tukey and Scheffe tests and p<0.05 was considered statistically significant. 

## Results

The weight of animals at the end of the experiment in SHE-treated group did not differ from the Con group. Regarding animal weight, no significant difference was observed between day 1 and day 30 (Table 1). 

Serum levels of estradiol and progesterone in SHE-treated group had no significant differences from those of the Con group (Table 1). 

The duration of the estrous cycle and its phases decreased in SHE-treated animals but they were not significantly different from those of the Con group (Table 2). 

**Figure 2:**
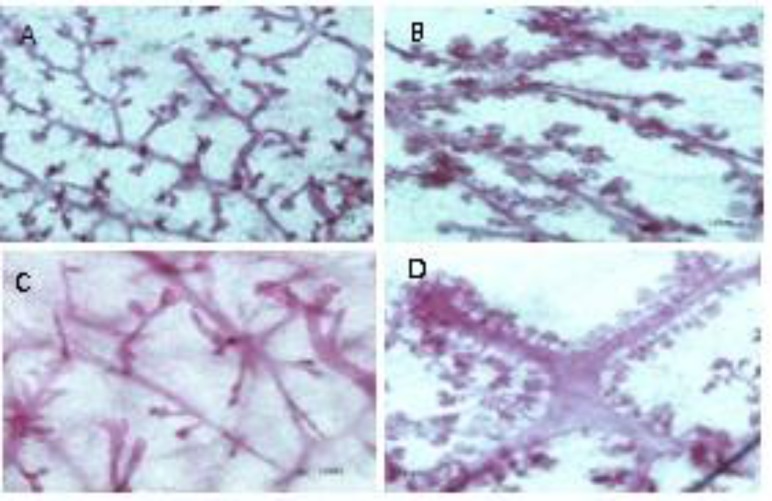
Photograph of mammary gland whole mounts of control (Con) group (A) and *S. officinalis* hydroalcoholic extract (SHE)-treated group (B). High magnifications of whole mount mammary gland of Con (C) and SHE (D) groups. Alveolar bud 1 and 2 and lobules increased in SHE-treated group. Carmine staining, scale bar=100μm

The number of AB1 and AB2 in three parts of mammary gland whole mounts increased significantly in SHE-treated group compared to the Con group. The number of AB1, AB2 and lobules in SHE-treated group were 1.25, 1.04 and 2 folds more than that of the Con group respectively (Table 3, Figure 2). Dif1 and Dif2 scoring values increased in SHE-treated group as compared to the Con group. However, significant difference was only seen in Dif1 value that was 1.42 folds higher in SHE-treated group as compared to the Con group (Table 3).

**Figure 3:**
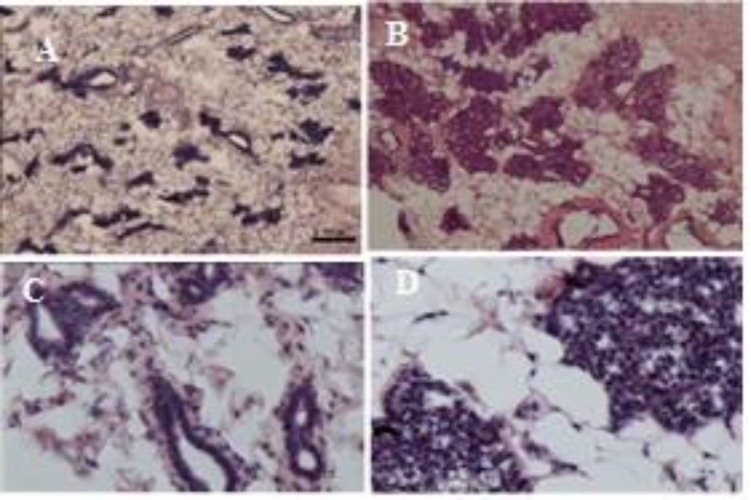
Transverse sections of mammary gland of the contol (Con) group (A) and *S. officinalis* hydroalcoholic extract (SHE)-treated group (B), scale bars=100 μm. Some ducts showed between the mammary glands stromal cells in the Con group but many alveolar buds collected toghter and consisted lobules among stromal cells in SHE-treated group. High magnifications of ducts (C) and lobules (D) in the Con and SHE groups, respectively, scale bars=10 μm. Hematoxyline-eosin staining

The number of ducts and lobules in mammary glands in histological sections from SHE-treated group increased significantly as compared to the Con group (Table 4 and Figure 3). Only a few diffused alveolar buds were observed as they formed many clusters or lobules (Figure 3-B). 

**Table 1:** Body weights and sex hormones concentrations of rats in the control group (Con) and *S. officinalis* hydroalcoholic extract (SHE)-treated group. Data showed as mean±SD.

Group	Body weight(g)	Increscent weight(g)	Estradiol concentration (pg/ml)	Progesterone concentration (pg/ml)
Con	187.40±10.38	18.28±8.65	52.63±12.66	1.95±1.63
SHE	178.86±10.03	17.60±10.24	56.78±23.74	2.86±2.05

**Table 2:** Duration of estrous cycle and its different phases (day) in the control group (Con) and *S. officinalis* hydroalcoholic extract (SHE)-treated group. Data showed as mean±SD

Group	Number of estrous cycle	Day of estrous cycle	Proestrus phase	Estrus phase	Diestrus phase
Con	6.75±0.95	26.50±3.51	6.10±0.58	5.41±1.00	14.90±2.64
SHE	5.86±0.38	23.31±4.46	5.62±0.79	5.20±0.95	12.48±3.64

**Table 3:** The alveolar buds (AB1 and AB2), lobules (L) and differentiations scoring (Dif 1 and Dif2) in mammary gland whole mounts of control group (Con) and *S. officinalis* hydroalcoholic extract (SHE)-treated group. Data showed as mean±SD.

Groups	AB1	AB2	L	Dif1	Dif2
Con	2.97±1.27	2.23±0.72	1.33±0.74	6.55±0.78	0.28±0.18
SHE	3.27±1.69*	2.34±1.00*	2.65±1.27*	9.29±1.03*	0.35±0.20

* Mean values were significantly different from the Con group (p<0·05). P values for AB1, AB2, L, Dif1, and Dif2 were 0.03, 0.001, 0.001, 0.001 and 0.13, respectively.

**Figure 4:**
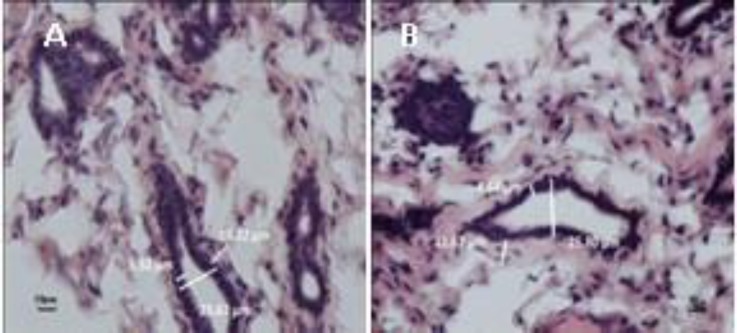
Histological sections of mammary glands in two groups of the Con (A) and *S. officinalis* hydroalcoholic extract (B). The ductal diameter of SHE-treated group was larger and ductal epithelium was shorter than that of the Con group. Hematoxyline-eosin staining. scale bars=10 μm

Ducts of mammary glands in SHE-treated group were dilated as compared to the Con group. In histological sections, ducts showed different shapes; therefore, we selected the same ducts from different groups but the widest parts of ducts in SHE-treated group were larger than those of the Con group (Figure 4). The epithelial diameters of ducts significantly decreased but the stromal layer around the ducts did not show any changes (Table 4 and Figure 4).

Periductul stroma of mammary glands in SHE-treated group revealed a higher number of mast cells as compared to the Con group. Some ducts in mammary glands contained secretions. The number of these ducts in SHE-treated group was higher than that of the Con group (Table 5).

**Table 4:** The mammary gland histomorphometrical changes of control (Con) and *S. officinalis* hydroalcoholic extract (SHE) treated groups in 5 microscopic fields of each section (three microscopic slides for each animal). Data showed as mean±SD

Groups	Lobules number	Ducts number	Ducts diameter (µm)	Ductal epithelial cell diameter (µm)	Ductal stromal diameter (µm)
Con	0.79±1.11	12.23±5.55	23.93±12.58	5.14±4.17	11.65±4.75
SHE	2.43±2.94*	32.14±30.4*	26.76±12.04*	4.37±1.16*	11.80±6.92

* Mean values were significantly different from Con group (p<0·05). P value for lobules number= 0.000, ducts number=0.000, ductus diamater= 0.002 and ductal epithelial cell diameter= 0.002.

**Table 5:** The mammary gland histomorphometrical changes in control (Con) and *S. officinalis* hydroalcoholic extract (SHE)-treated groups in five microscopic fields of each part. Data showed as mean±SD.

Groups	Number of ductal branch	Number of mast cells	Number of ducts containing secretion
Con	0.14±0.40	1.69±1.31	0.09±0.38
SHE	0.24±0.60	2.14±1.54*	0.62±1.13*

* Mean values were significantly different from Con group (p< 0·05). P value for number of mast cells= 0.000 and number of ducts containing secretion= 0.000.

## Discussion

The weight of SHE-treated rats did not show any significant difference as compared to the Con group. Therefore, it may be concluded that *S. officinalis* has no side effects on body growth.

Normally, estrous cycle in rat lasts for 4-5 days. Hormonal alterations can cause estrous cycle irregularity. According to our results, estrous cycle and its phases and estradiol and progesterone concentrations did not change significantly after SHE treatment. Although sage contained some estrogenic components, it did not interfere with hormonal balance. 

According to our data, the number of AB1 and AB2 in three parts of mammary glands whole mounts, Dif1 and Dif2 values, and the number of ducts, lobules and branching ducts of mammary glands in histological sections of SHE-treated group increased significantly as compared to the Con group. Alveoli of adults mammary gland are mainly secreted in pregnancy, when rising levels of prolactin, estrogen, and progesterone cause further ductual branching, accompanying an increase in adipose tissue and a higher blood flow. *S. officinalis* hydroalcoholic extract promoted the growth of the mammary glands alveoli of rats similar to the effect of high levels of steroids hormones. These effects could be attributed to estrogenic components of sage. There are two main types of phytoestrogens namely isoflavones and lignans. Isoflavones are generally absorbed and can be detected in urine. Sage is a natural source of flavonoids and polyphenolic compounds (e.g. carnosic acid, rosmarinic acid and caffeic acid). Carnosic acid and rosmarinic acid are present at high concentrations in the extract of sage plants (Yurtseven et al., 2008 ▶). Phytoestrogens are nonsteroidal component of plants that attach to alpha and beta estrogen receptors and induce biological effects in the way similar to natural estrogens such as 17β-estradiol, (Ososki and Kennelly, 2003 ▶). Phytoestrogens show either agonistic or antagonistic effects based on estrogen level of serum and estrogenic receptors saturation (Adlercreutz et al., 1993 ▶). When estrogen level in blood is low, phytoestrogens have agonistic potential and show effects similar to those of estrogen and vice versa (Kurzer and Xiz, 1997 ▶). Nelson et al., reported that phytoestrogens have the same effects as the estrogen on mammary gland proliferation and differentiation (Nelson et al., 1992 ▶). Phytoestrogens have been shown to influence the growth and division of vaginal epithelium and alveolar epithelial cells of mammary gland in postmenopausal women (Wilcox et al., 1990 ▶; Baird et al., 1995 ▶; Murkies et al., 1995 ▶; Petrakis et al., 1996 ▶; Albertazzi et al., 1998 ▶). Estrogen and progesterone receptors are distributed all over the epithelial, adipose, and connective tissue components of normal mammary gland in rodents (Edery et al., 1985 ▶; Haslam and Shyamala, 1981 ▶). Immunohistochemical studies of mice treated with genistein (a phytoestrogen) revealed an increase in the level of alpha and beta estrogen receptors mRNA which can lead to mammary gland growth (Padilla-Banks et al., 2006 ▶).

The number of mast cells (one of the stromal cells) of mammary glands of SHE-treated rats increased significantly as compared to the Con group. It may be due to either estrogenic activity of sage extract on stromal cells or anti-allergic response of stromal cells against antigenic activity of extract components. Mast cells increase in stromal microenvironment of mammary gland at puberty and breast morphogenesis when its alveolar buds are proliferated and its ducts are branched (Lilla and Werb, 2010 ▶). 

Lumens of ducts in mammary glands of SHE-treated group were dilated and had much secretion as compared to the Con group. It may be related to high activity of alveoli epithelial cells when they are exposed to sage phytoestrogens. This secretion can be due to sage proteins aggregation in lumen, as well. Stimulatory effect of soy proteins (as a plant with a high content of phytoestrogens) on glandular secretion of breast in pre-and post-menopausal women have been reported (Petrakis et al., 1996 ▶). 

*S. officinalis* hydroalcoholic extract, just similar to steroids hormones stimulated the growth of mammary gland alveolar buds and ducts and induced lobule formation in adult female rats. This medicinal herb showed no side effects on body weight, estrous cycle duration and sex hormones concentrations. Therefore, it can be considered as a lactiferous herb. 
